# Individual responses to pomegranate juice on recovery from exercise-induced muscle damage in collegiate male volleyball players

**DOI:** 10.1080/15502783.2026.2642149

**Published:** 2026-04-21

**Authors:** Gholamreza Rezaei, Mohammad Hemmatinafar, Mark E.T. Willems, Dena Mastouri, Babak Imanian, Rasoul Rezaei

**Affiliations:** a Department of Sport Sciences, Faculty of Education and Psychology, Shiraz University, Shiraz, Iran; b School of Sport, Science and Engineering, University of Chichester, Chichester, United Kingdom; c Department of Community Nutrition, School of Nutritional Sciences and Dietetics, Tehran University of Medical Sciences, Tehran, Iran

**Keywords:** Pomegranate juice, exercise-induced muscle damage, delayed-onset muscle soreness, volleyball, smallest worthwhile change, recovery

## Abstract

**Background:**

Volleyball demands frequent explosive, stretch–shortening muscle actions that elevate the risk for exercise-induced muscle damage (EIMD) and delayed-onset muscle soreness (DOMS). Polyphenol-rich pomegranate juice (POMj) has been proposed to aid recovery, yet evidence in highly trained team-sport athletes is limited. This study investigated whether short-term POMj enhances functional and isokinetic recovery following an EIMD protocol in collegiate male volleyball players and identified individual responders using the smallest worthwhile change (SWC) method.

**Methods:**

Fourteen Tier-3 male collegiate volleyball players completed testing in a randomized, double-blind, placebo-controlled crossover design. The supplementation was 1000 mL of natural POMj (500 mL evening prior; 500 mL 2 h pre-EIMD protocol) or flavor-matched placebo (PLA). The EIMD protocol consisted of 200 weighted (10% body mass) maximal vertical jumps. The outcomes at baseline (BL) and 48 h post-EIMD included functional tests (vertical jump height, handgrip, medicine-ball throw, flexibility, wall-squat) and knee isokinetic/isometric contraction indices (extension/flexion at 30° s^−1^ and 180° s^−1^; MVIC at 45°). DOMS (VAS) was assessed at BL, 0, 12, 24, and 48 h. Repeated-measures ANOVA was used to test condition effects. The SWC was set to 0.2 × SD at BL to classify individual responders.

**Results:**

ANOVA revealed a significant main effect of time for most variables (*p *< 0.05), indicating recovery or changes across 48 h; however, no significant between-condition differences (POMj vs. PLA) were observed for any functional or isokinetic parameter. Although the knee extensor peak torque at 180° s^−1^ significantly improved from baseline within the POMj (*p *= 0.002), this recovery was not statistically superior to that of the PLA (*p *= 1.000). Similarly, DOMS increased significantly over time (*p* = 0.001), with no significant difference between conditions. SWC analysis revealed higher responder proportions with POMj for selected 180° s^−1^ indices (e.g. knee extensor relative peak torque (RPT): ~86% POMj vs. ~64% PLA; absolute peak torque (APT): ~79% vs. ~71%), indicating practically meaningful individual benefits despite null mean differences.

**Conclusions:**

In highly trained male volleyball athletes, short-term POMj did not outperform placebo on group-mean recovery outcomes at 48 h post-EIMD; however, SWC-based analyses showed a higher proportion of meaningful responders with POMj in selected high-velocity isokinetic measures, supporting responder-focused evaluations of recovery nutraceuticals in sport-specific settings.

## 1. Introduction

Volleyball is characterized by frequent movements with explosive stretch–shortening muscle actions—such as repeated jumping, blocking, and spiking—that impose substantial eccentric loads on the lower limbs and shoulder girdle. Performance and match-analysis studies have shown that elite players execute a high number of maximal or near-maximal jumps and spikes per match, placing considerable mechanical stress on the neuromuscular system [1]. These repeated stretch–shortening muscle actions can induce substantial exercise-induced muscle damage (EIMD), manifested by microtrauma and delayed-onset muscle soreness (DOMS), and contribute to a high incidence of overuse concerns in players, particularly because neuromuscular function and strength often remain depressed for ≥72 h after a single bout of muscle-damaging exercise [2]. More generally, high-intensity or unaccustomed exercise often leads to EIMD, especially when eccentric muscle contractions are involved [3]. EIMD is characterized by microscopic myofibrillar disruptions followed by a secondary inflammatory response as leukocytes infiltrate damaged tissue, with clinical and functional sequelae, including DOMS, swelling, reduced muscle function, and leakage of muscle enzymes such as creatine kinase into the bloodstream [3,4]. These symptoms typically emerge within the first day after unaccustomed eccentric exercise, peak around 24–48 h, and can remain elevated for several days, with soreness and functional impairments often still evident up to 5–7 days postexercise [5]. While EIMD-related inflammation is part of the normal exercise response, excessive damage can temporarily impair performance capacity; experimental studies have shown that DOMS and the associated loss of strength typically persist for several days and, in more severe cases, can remain evident for a week or longer, during which athletes may struggle to maintain usual training intensity or competitive performance [5,6]. Consequently, reducing the severity and duration of EIMD/DOMS has become a practical priority to sustain high-quality training sessions and rapid competitive turnaround [5]. A range of strategies—from compression garments to nutritional interventions—are commonly employed to alleviate symptoms and accelerate the recovery of full muscle function [7,8].

Among nutritional approaches, polyphenol-rich supplements have gained increasing attention as potential strategies to mitigate EIMD and DOMS, with several trials reporting reduced soreness and improved strength recovery after muscle-damaging exercise [8–10]. Pomegranate juice (POMj), derived from *Punica granatum*, is a rich dietary source of polyphenols, particularly ellagitannins and anthocyanins, which are metabolized by the human gut microbiota into bioactive urolithin derivatives and contribute to the antioxidant and anti-inflammatory effects observed with POMj consumption [11,12]. Clinical trials have demonstrated that regular consumption of POMj is associated with reduced oxidative stress and improvements in cardiovascular risk markers, including blood pressure and vascular function [13]. In the context of exercise recovery, recent studies suggest that POMj supplementation can mitigate some of the detrimental effects of EIMD. For example, consuming POMj in the days surrounding intense exercise has been shown to elevate blood antioxidant capacity and lower biomarkers of oxidative damage after resistance exercise [14]. These biochemical changes correspond to functional benefits: POMj groups often exhibit faster recovery of muscle strength and power than placebo. In nonresistance-trained men, once- or twice-daily natural POMj taken for several days surrounding unaccustomed eccentric exercise improved isometric strength recovery in both elbow flexors and knee extensors, with no between-group differences in muscle soreness while performing one repetition [15]. Similarly, trained athletes who were supplemented with POMj experienced reduced DOMS and enhanced strength restoration in upper-body muscles following damaging exercise [16]. Such findings suggest that pomegranate polyphenols aid muscle repair, possibly by blunting excessive inflammation and oxidative damage that delay recovery [17]. Another potential mechanism is improved hemodynamics: acute POMj or extract intake can increase blood vessel diameter and blood flow postexercise [18], likely via enhanced nitric oxide bioavailability. This vasodilatory effect could promote nutrient delivery and waste removal in muscles, thereby facilitating recovery, although its direct impact on performance restoration remains to be confirmed [18]. Effective dosing protocols in the literature typically range from 500 mL of POMj (providing ~650 mg of polyphenols) daily for ~5 days before exercise to acute high-polyphenol doses shortly before exercise [18,19]. Overall, the current evidence points to POMj as a promising natural intervention to speed recovery from muscle-damaging exercise via antioxidant and anti-inflammatory pathways, as well as possible perfusion benefits [15,18].

Despite these encouraging results, research on POMj for exercise recovery is still emerging, and some gaps remain. Not all studies have found uniform benefits across all outcomes; for instance, specific trials noted significant improvements in strength recovery with POMj but no concomitant reduction in muscle soreness [14]. The efficacy of POMj may depend on the context, as exercise mode, muscle groups, and training status of participants are likely moderating factors [15,17,20]. Most POMj studies to date have focused on isolated eccentric exercises in laboratory settings (e.g. single-joint resistance tasks) and often with untrained or recreational subjects [15,17]. Fewer data are available for highly trained athletes or team-sport scenarios that involve repeated high-intensity efforts and multijoint movements. Indeed, a recent review emphasized that while POMj appears effective for recovery from resistance-based EIMD, its effects following endurance exercise or sport-specific protocols remain unclear [21]. Moreover, well-trained individuals may exhibit a blunted damage response, potentially reducing the observable benefit of antioxidants unless a sufficiently stressful protocol is used [22]. These considerations underscore the need for further research on pomegranate supplementation in athletic populations under ecologically valid conditions. By studying POMj in competitive athletes and using more comprehensive performance tests, we can determine whether its recovery benefits translate to real-world sports settings and rigorous exercise demands.

To evaluate EIMD interventions meaningfully, outcomes should prioritize functional performance (strength, power, neuromuscular function) rather than relying solely on muscle soreness ratings or biochemical markers. Postdamage decreases in jump height, sprint speed, and maximal strength can persist for 24–72+ h and directly impair sport performance; thus, tests such as isometric/isokinetic strength, vertical jump, and sprint/agility best capture practical recovery [23]. Notably, functional measures can reveal benefits not reflected in muscle soreness ratings; for example, in resistance-trained men, POMj improved elbow flexor strength recovery and reduced muscle soreness, whereas knee extensor strength and muscle soreness were unchanged compared with placebo—underscoring the value of performance endpoints [17]. For team-sport athletes such as volleyball players, emphasizing strength, explosive power, and neuromuscular testing offers real-world recovery benefits that help athletes return to optimal training and competition levels.

The smallest worthwhile change (SWC) provides an athlete-centered lens for interpreting performance, defining a meaningful improvement as a small effect (≈Cohen's *d* 0.2; commonly 0.2 × SD of baseline/control) that exceeds normal day-to-day variability [24]. By focusing on individual responses, SWC complements group statistics and helps reveal heterogeneity that traditional cohort analyses may mask [25], a feature particularly valuable in nutrition studies with high interindividual variability [24]. Recent applications illustrate this utility: in strength-trained men, Teimouri-Korani et al. identified sizeable proportions of responders to caffeine using SWC despite modest group means [26]; in elite soccer players, Niknam et al. showed that a polyphenol-containing purple grape juice produced above-SWC gains in key endurance and explosive-power outcomes even when group averages were nonsignificant [27]. To date, SWC-based analyses with POMj are scarce; therefore, applying SWC in the present trial addresses this gap by quantifying practically meaningful, individual-level recovery benefits alongside conventional statistics.

EIMD and DOMS impede athletes' capacity to sustain high-quality training, and emerging evidence indicates that POMj may aid recovery by improving antioxidant status, attenuating strength loss, and, in some cases, reducing soreness after eccentric exercise [7,14]. However, data on highly trained team-sport athletes performing functional, multijoint tasks remain limited. To address this gap, we conducted a randomized, double-blind, placebo-controlled crossover trial in collegiate male volleyball athletes. This design enables each participant to serve as their own control, thereby strengthening inference by reducing interindividual variability. Primary endpoints emphasize performance-based outcomes (strength, power, and functional mobility) rather than solely subjective markers, directly testing the practical recovery value of POMj. We also apply the SWC to quantify the proportion of athletes who achieve meaningful individual improvements compared with placebo. By combining SWC with conventional analyses, this study provides a rigorous evaluation of POMj's efficacy in a sport-specific context, aiming to inform athlete-centered recovery strategies in training and competition.

## 2. Methodology

### 2.1. Participants

Fourteen male collegiate volleyball players with an average of three years of competitive league experience were recruited for this study. According to the classification system of McKay et al., all athletes were categorized as Tier 3 (highly trained/national level) [28]. The descriptive characteristics of the participants (age, height, and body mass; mean ± SD) are presented in Table 1. Eligibility was determined using health and exercise history questionnaires. The inclusion criteria required a minimum of three years of volleyball experience, the absence of any self-reported allergy or intolerance to pomegranate juice, and a habitual sleep duration of 7–8 h per 24-h period. During the data collection period, participants reported abstaining from smoking, alcohol consumption, and beverages containing nitrates or caffeine. All the athletes were enrolled in the same training camp and followed a standardized training program under the supervision of their team coaches. To minimize potential confounding from prior fatigue, they were instructed to refrain from strenuous physical activity for 48 h before and after each intervention session. The study was conducted approximately two months before the start of the competitive volleyball league season to increase the relevance of the findings to preseason conditioning and recovery strategies. The study protocol was explained in detail to all participants, and written informed consent was obtained prior to enrollment. The procedures conformed to the principles of the Declaration of Helsinki and were approved by the Human Research Ethics Committee of Shiraz University (IR.US.PSYEDU.REC.1404.032).

**Table 1.** Characteristics of participants (*n *= 14).

Characteristic	Mean ± SD
Age (year)	22 ± 2
Height (cm)	185 ± 8
Weight (kg)	73 ± 6

### 2.2. Sample size calculation and study design

An a priori sample size calculation was performed using G Power software (version 3.1.9.7) [29] for a repeated-measures ANOVA (within-subjects factor). The analysis assumed a significance level of *α *= 0.05, a desired statistical power (1 − *β*) = 0.80, an assumed correlation among repeated measures of *r* = 0.90, and no sphericity correction (*ε *= 1). The expected effect size was derived from previous research on the efficacy of polyphenol-rich pomegranate juice in restoring muscle function and strength. Specifically, based on standardized improvements in isometric strength and peak torque recovery reported in similar intervention protocols [17], a moderate-to-large effect size of *d* = 0.60 was anticipated. For use in the repeated-measures ANOVA framework, this value was converted to Cohen's f using the approximate relation *d* ≈ 2f*, yielding an effect size of *f* = 0.30. With these parameters (effect size *f* = 0.30, *α *= 0.05, power = 0.80, two measurements, correlation = 0.90, *ε *= 1), G*Power indicated a required total sample size of *n *= 12 participants. To account for possible attrition and increase the robustness of the analyses, the target sample size was increased to 14 participants, all of whom completed the study.

**Figure 1.**
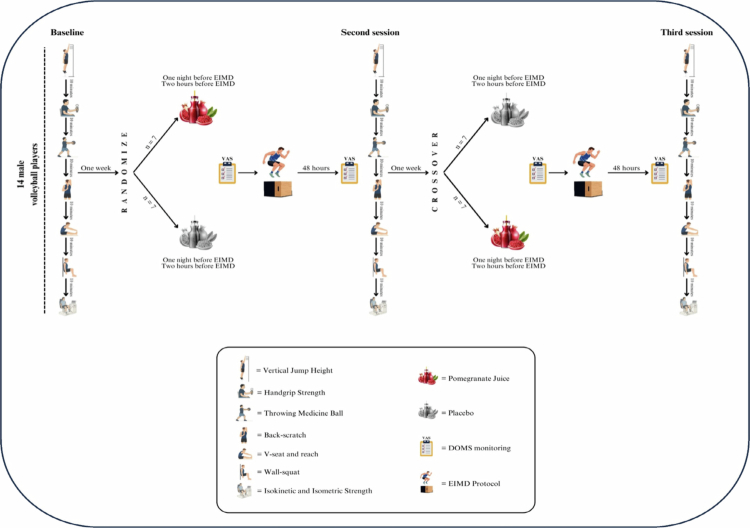
The protocol of taking supplements and performing tests during the three conditions. VAS: visual analog scale, EIMD: exercise-induced muscle damage.

**Figure 2.**
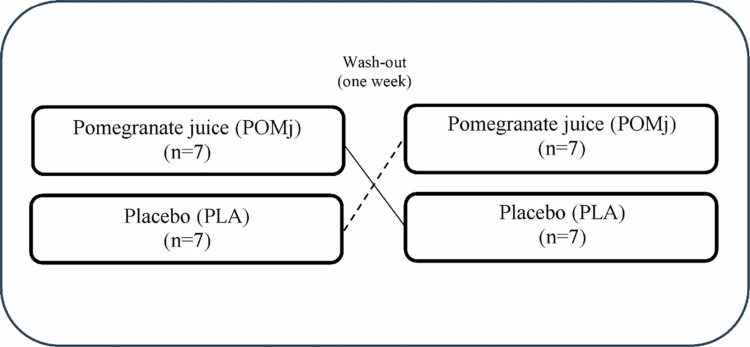
The randomization process of the crossover design.

This randomized, double-blind, placebo-controlled, crossover study investigated the effects of POMj on post-EIMD recovery of performance and muscle soreness in collegiate male volleyball players, following published guidelines analysis of interindividual differences [24] and established statistical perspectives for individual responses in exercise science [30,31]. To enhance protocol familiarity and reduce interindividual variability, athletes completed two preliminary sessions to learn all testing procedures, including Biodex dynamometry and the EIMD protocol adapted from prior work [32]. At baseline (BL), before any supplementation, recordings were made for vertical jump height (VJH), handgrip strength (HGS), backward overhead medicine-ball throw (TMB), back-scratch (BS), V-seat reach, wall-squat, and knee extensor/flexor strength (isokinetic at 30° s^−1^ and 180° s^−1^; maximum voluntary isometric contraction, MVIC, at 45°). A 10-min active rest separated tests (both functional and strength tests) were performed to limit fatigue carryover. One week later, the participants were randomized in a double-blind manner to receive POMj or a placebo (PLA). The supplementation totaled 1000 mL: 500 mL the evening before the EIMD protocol and 500 mL 2 h pre-EIMD protocol. DOMS was assessed via a 10-cm visual analogue scale (VAS) at BL, immediately post-EIMD, and at 12, 24, and 48 h [33]. At 48 h post-EIMD, all the functional and strength tests were repeated. After a 7-day washout, the athletes crossed over to the alternate condition, and the protocol was replicated to mitigate period and carryover effects (Figures 1 and 2). Standardization measures were implemented to maximize internal validity: a unified warm-up before each visit; a standardized breakfast 90 min presession (~250 kcal; 45 g carbohydrate, 9 g protein, 5 g fat) [34]; and written dietary guidance to maintain habitual intake while avoiding polyphenol-rich foods/beverages (e.g. berries, grape juice, tea/coffee, dark chocolate), caffeine, nitrate-rich vegetables (e.g. beetroot, spinach), and alcohol for 48 h pretesting. Athletes were asked to refrain from strenuous activity for 48 h before and after each intervention session. All trials were conducted between 10:00 and 12:00 to minimize diurnal variation. These procedures were designed to enhance reliability and ensure that any observed effects could reasonably be attributed to the POMj intervention.

### 2.3. Randomization and blinding

Participants were assigned 1:1 to two sequences, PLA → POMj or POMj → PLA, using a computer-generated random list with concealed allocation (sequentially numbered, opaque, sealed envelopes). Beverages were flavor- and color-matched, coded by an independent custodian, and investigators, athletes, and assessors were blinded throughout. The crossover was fully counterbalanced (seven athletes per sequence) with a 7-day washout to limit carryover, controlling for order/period effects consistent with Latin-square principles [35]. Unblinding occurred only after data lock.

### 2.4. Supplementation procedures

Following prior protocols [8,36], athletes received 1000 mL of natural POMj prepared from whole fruit 48 h before data collection and then frozen at −4 °C until use. A sample was analyzed via high-performance liquid chromatography (HPLC) to determine the polyphenolic profile. The composition is shown in Table 2; no additives were included. The dose was 500 mL the evening before and 500 mL 2 h before the EIMD session. The PLA was a pomegranate-flavored beverage (water, citric acid, natural/nature-identical flavorings, sweeteners: aspartame 0.3 g L^−1^, acesulfame-K 0.16 g L^−1^; stabilizer: Arabic gum) verified to be free of antioxidants, vitamins, and polyphenols. Beverages were provided in identical containers to maintain blinding. No adverse events were observed.

**Table 2.** Polyphenolic composition of 1000 ml of the pomegranate juice determined via HPLC analysis.

Polyphenol	mg/L (PPM)
Tannic acid	1557.13
Benzoic acid	34.19
Chlorogenic acid	31.79
Syringic acid	28.40
Ferulic acid	5.23
Gallic acid	17.61
Vanillic acid	17.82
Ellagic acid	16.39
P-coumaric acid	1.79
Rutin	34.37
**Total**	**1744.72**

### 2.5. Exercise-induced muscle damage protocol

The participants first completed a standardized 10-min warm-up (dynamic mobility, light jogging, and stretching). Muscle damage was then induced with 200 maximal vertical jumps performed while wearing a weighted vest equal to 10% body mass: 10 sets × 20 jumps, executed at a cadence of one jump every 4 s, with 2-min seated rest between sets. This protocol was adapted from our previously validated dietary supplementation studies [31,32,37,38]. Throughout the intervention, all the athletes trained within the same camp and followed an identical coach-supervised program to maintain consistent external training loads.

### 2.6. Examination of delayed-onset muscle soreness by the VAS scale

DOMS was assessed using a 10-cm visual analog scale (VAS), a validated tool for experimental and clinical pain evaluation [39]. The VAS consisted of a horizontal line anchored by “no pain” (0 mm) on the left and “severe pain” (100 mm) on the right. At each time point—baseline (BL), immediately post-EIMD (0 h), 12, 24, and 48 h—participants marked their perceived soreness on the line. Scores were obtained by measuring the distance from the left anchor to the mark with a ruler and were recorded in millimeters [33]. The selected time points reflect the typical DOMS trajectory after high-intensity eccentric exercise—onset ≈12 h, peak ~24–72 h, and gradual resolution thereafter—per prior literature [40]. This schedule enabled a consistent, time-course evaluation of DOMS across the intervention.

### 2.7. Functional tests

Vertical jump height (VJH): The Sargent Jump Test, also known as the vertical jump test, is a widely used assessment of lower-limb explosive strength. Developed by Dr. Dudley Allen Sargent in the early 20th century, this test evaluates an individual's ability to perform a vertical leap from a standstill position [41]. During the test, the participants applied chalk to their fingertips, stood close to a wall, and reached up to mark it with the tip of their fingers (M_1_). They jumped as high as possible from a static position, marking the wall again (M_2_). VJH was calculated as the distance between M_1_ and M_2_. The test was repeated three times, with a 1-min passive rest between attempts, and the highest jump was used for analysis [42].

Handgrip strength (HGS): hand grip strength, used as an index of upper-body isometric force and neuromuscular function, was assessed with a calibrated hydraulic hand dynamometer (Saehan SH5001; SAEHAN Corporation, Changwon, Republic of Korea; capacity 200 lbf/90 kg). The participants were seated with their elbow at 90° flexion; measurements were obtained for the dominant hand. After receiving standardized instructions, each participant performed three maximal isometric efforts per hand with a 1-min rest between trials; the highest value was retained for analysis. The testing order was randomized, and participants were instructed to discontinue if any pain occurred. The procedures followed were in accordance with established guidelines for isometric strength assessment in exercise science [43].

Backward overhead throwing medicine ball (TMB): Upper- and whole-body explosive power were assessed with the standing backward overhead throwing medicine ball using a rubber medicine ball weighing 3 kg with a diameter of approximately 22 cm [44]. Athletes stood on a marked line, feet shoulder-width apart and held the ball with both hands. They performed a backward overhead swing and were instructed to release the ball with maximal effort. Slight countermovement was permitted, but both feet remained in contact with the floor. Each player completed three trials; the greatest horizontal distance from the line to the first point of ground contact was recorded for analysis. The TMB provides a reliable index of total-body explosive strength, with an emphasis on the posterior chain, core, and upper-body coordination, and is functionally relevant to volleyball actions that require rapid trunk and upper-limb power.

Back-scratch (BS): The back-scratch test [45] was used to assess upper-body flexibility. The test consists of measuring the overall shoulder range of motion by measuring the distance between (or overlap of) the middle fingers as they come together behind the back. This test was performed twice with both hands; alternatively, the final score in millimeters (mm) was calculated as the mean value of the best attempts for both arms.

V-seat and reach (V-seat): Flexibility was assessed with the V-sit and reach test [46]. Two 2-m lines were marked on the floor at 45°, meeting at the wall; a tape measure was placed perpendicular to the wall with 0 cm at the apex. The participants sat barefoot with their legs aligned to the lines (~45° abduction), with their knees fully extended, and with their backs/heads against the wall at the beginning. With their hands overlapped, palms down, they reached forward slowly, allowing their fingers to slide along the tape while their knees remained extended. Each athlete performed three trials; the best distance (cm) from the starting mark to maximal reach was recorded for analysis.

Wall-squat: Lower-body endurance was assessed with the wall-squat. The players stood with their backs and shoulders flat against the wall and feet shoulder width apart and descended to 90° at the knees and hips; the arms hung naturally. They held the position until failure—defined as inability to maintain joint angles, heel lift, trunk shift, or loss of back/shoulder contact—with no verbal encouragement. The time to exhaustion (s) was recorded. Familiarization preceded testing to standardize the technique. The wall squat is a measurement of the isometric endurance of the quadriceps, hamstrings, and gluteals, supporting postural stability in repeated volleyball actions [37,47,48].

Isokinetic and isometric strength tests: Knee extensor/flexor strength of the dominant leg was assessed on a Biodex System 4 Pro (Biodex Medical Systems, Shirley, NY, USA) in the concentric–concentric mode at 30° s^−1^ and 180° s^−1^. After positioning and gravity correction (Biodex software), the participants were stabilized with straps (chest, pelvis, thigh, and above the ankle). The knee's estimated transverse axis was aligned with the dynamometer axis; ROM = 80°. Each velocity comprised five maximal repetitions (extension–flexion) with 60 s of rest between sets. The outcomes included absolute peak torque (APT), relative peak torque (RPT), time to peak torque (TPT), average rate of force development (AvRFD = APT/TPT), and average power (AvP) [49,50]. Velocity selection was intentional: 30° s^−1^ assessed maximal strength under tightly controlled speed, whereas 180° s^−1^ evaluated rapid force generation and power relevant to sport-specific actions [26,51,52].

Maximum voluntary isometric contraction (MVIC) was measured at a 45° knee angle (both extension and flexion) using the same device after 10 min of active rest. The participants performed five 5-s maximal efforts separated by 60 s of rest, with standardized stabilization and alignment as above. A value of 45° was chosen to reflect a joint position with reliable near-peak torque from favorable length–tension relationships while limiting joint strain [53].

### 2.8. Statistical analysis

Analyses were performed in SPSS v28 (IBM, Chicago, IL, USA). Data normality was checked with the Shapiro–Wilk test. Primary outcomes (isokinetic/isometric indices and functional tests) were evaluated with a two-way repeated-measures ANOVA with factor conditions (POMj, PLA) and time (e.g. BL, post-EIMD time points); when sphericity was violated (Mauchly's test), Greenhouse–Geisser corrections were applied. Bonferroni-adjusted pairwise comparisons probed significant effects. To profile individual responses, the smallest worthwhile change (SWC) was computed as 0.2 × SD at baseline (BL), and athletes whose change exceeded the SWC threshold within each condition were classified as responders [25,26]. Partial eta squared (*ηp*²) indexed effect size, interpreted as small ≥0.01, medium ≥0.06, or large ≥0.14 [54]. Statistical significance was set at *p* ≤ 0.05. The results are reported as mean ± SD. Figures were created in GraphPad Prism v9.0.0 (GraphPad Software, San Diego, CA, USA).

## 3. Results

**Figure 3.**
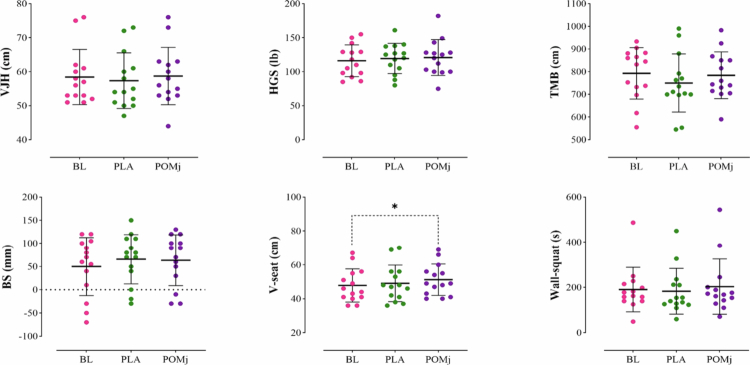
Individual responses, means, and standard deviations of the functional tests (VJH, HGS, TMB, BS, V-seat, and Wall-squat) in the three conditions. BL: baseline, PLA: placebo, POMj: pomegranate juice, VJH: vertical jump height, HGS: handgrip strength, TMB: throwing medicine ball, and BS: back-scratch. *Significant difference compared to the BL (*p *< 0.05).

**Figure 4.**
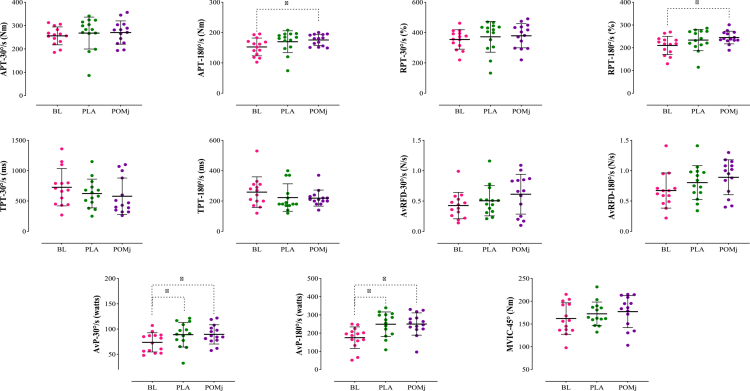
Individual responses, means, and standard deviations of the knee extensor isokinetic (APT, RPT, TPT, AvRFD, and AvP at 30°/s and 180°/s) and isometric (MVIC at 45°) parameters under the three conditions. BL: baseline, PLA: placebo, POMj: pomegranate juice, APT: absolute peak torque, RPT: relative peak torque, TPT: time to peak torque, AvRFD: average rate of force development, AvP: average power, and MVIC: maximum voluntary isometric contraction. *Significant difference compared to the BL (*p *< 0.05).

**Figure 5.**
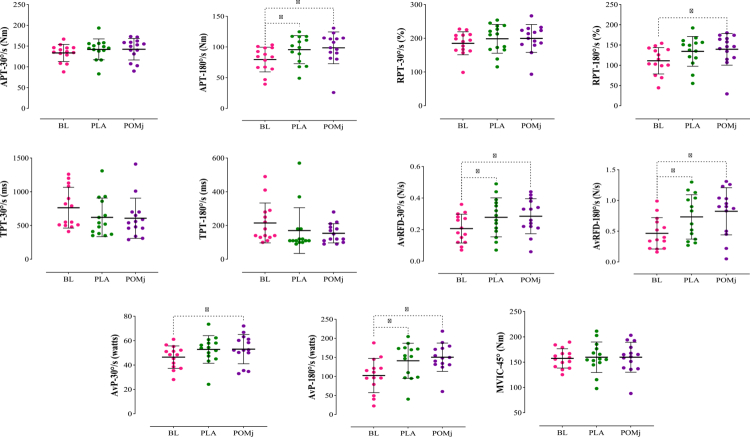
Individual responses, means, and standard deviations of the knee flexor isokinetic (APT, RPT, TPT, AvRFD, and AvP at 30°/s and 180°/s) and isometric (MVIC at 45°) parameters under the three conditions. BL: baseline, PLA: placebo, POMj: pomegranate juice, APT: absolute peak torque, RPT: relative peak torque, TPT: time to peak torque, AvRFD: average rate of force development, AvP: average power, MVIC: maximum voluntary isometric contraction. *Significant difference compared to the BL treatment (*p *< 0.05).

**Figure 6.**
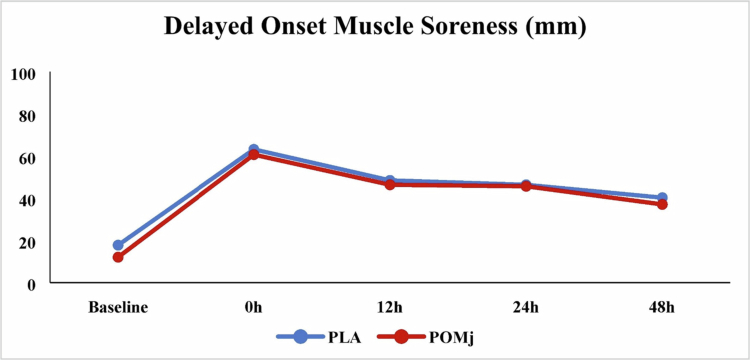
Average DOMS under various supplementation conditions. PLA: placebo, POMj: pomegranate juice, Baseline: before the EIMD; 0 h: immediately after the EIMD; 12 h: 12 hours after the EIMD; 24 h: 24 hours after the EIMD; and 48 h: 48 hours after the EIMD. “Significant differences” are mentioned in the results section.

Group descriptive statistics (means ± SD and % change) for functional tests (VJH, HGS, TMB, BS, V-sit, wall-squat), isokinetic knee indices (APT, RPT, TPT, AvRFD, and AvP at 30° s^−1^ and 180° s^−1^), and isometric strength (MVIC at 45°) are summarized in Table 3; full inferential outputs appear in Table 4, with individual-response plots in Figures 3–5 and the DOMS time course in Figure 6.

**Table 3.** Means, standard deviation (SD), and relative changes (%RC) of the variables in the three conditions (*n *= 14).

	Variables	BL	PLA	POMj	%RC_PLA/BL_	%RC_POMj/BL_	%RC_POMj/PLA_
Functional	VJH (cm)	58 ± 8	57 ± 8	58 ± 8	−1.8%	0.5%	2.3%
	HGS (lb)	116 ± 23	119 ± 22	120 ± 26	2.8%	4.0%	1.2%
	TMB (cm)	792 ± 113	750 ± 128	784 ± 103	−5.3%	−1.0%	4.5%
	BS (mm)	50 ± 62	62 ± 61	63 ± 54	24.2%	27.0%	2.2%
	V-seat (cm)	47 ± 9	49 ± 10	51 ± 9	2.7%	7.1%	4.3%
	Wall-squat (s)	190 ± 98	183 ± 101	217 ± 168	−3.9%	14.2%	18.8%
Knee extensors	APT-30°/s (Nm)	256 ± 38	267 ± 68	270 ± 49	4.6%	5.5%	0.8%
	APT-180°/s (Nm)	152 ± 29	169 ± 36	175 ± 18	11.3%	15.1%	3.4%
	RPT-30°/s (%)	354 ± 64	371 ± 101	379 ± 79	4.8%	6.9%	1.9%
	RPT-180°/s (%)	210 ± 40	233 ± 46	244 ± 27	11.2%	16.5%	4.7%
	TPT-30°/s (ms)	727 ± 307	623 ± 237	578 ± 300	−14.2%	−20.4%	−7.2%
	TPT-180°/s (ms)	258 ± 100	222 ± 90	219 ± 52	−13.8%	−15.2%	−1.6%
	AvRFD-30°/s (N/s)	0.42 ± 0.21	0.50 ± 0.24	0.61 ± 0.32	19.0%	45.2%	22.0%
	AvRFD-180°/s (N/s)	0.67 ± 0.28	0.80 ± 0.27	0.89 ± 0.28	19.4%	32.8%	11.2%
	AvP-30°/s (watts)	73 ± 18	88 ± 24	89 ± 19	20.6%	21.5%	0.7%
	AvP-180°/s (watts)	174 ± 59	248 ± 67	249 ± 61	42.5%	43.0%	0.3%
	MVIC-45° (Nm)	161 ± 34	172 ± 25	177 ± 35	6.3%	9.3%	2.8%
Knee flexors	APT-30°/s (Nm)	133 ± 20	142 ± 25	142 ± 25	6.3%	6.6%	0.2%
	APT-180°/s (Nm)	79 ± 20	95 ± 22	98 ± 28	20.3%	24.0%	3.1%
	RPT-30°/s (%)	185 ± 34	198 ± 42	199 ± 41	7.2%	7.8%	0.6%
	RPT-180°/s (%)	110 ± 32	134 ± 36	139 ± 39	20.9%	25.7%	3.9%
	TPT-30°/s (ms)	763 ± 302	620 ± 286	607 ± 297	−18.7%	−20.4%	−2.1%
	TPT-180°/s (ms)	215 ± 118	170 ± 135	153 ± 56	−20.9%	−28.5%	−9.6%
	AvRFD-30°/s (N/s)	0.20 ± 0.09	0.27 ± 0.12	0.28 ± 0.11	35.0%	40.0%	3.7%
	AvRFD-180°/s (N/s)	0.46 ± 0.25	0.73 ± 0.36	0.82 ± 0.38	58.7%	78.2%	12.3%
	AvP-30°/s (watts)	46 ± 9	52 ± 11	53 ± 11	13.5%	14.0%	0.4%
	AvP-180°/s (watts)	102 ± 44	141 ± 46	150 ± 37	37.8%	47.0%	6.6%
	MVIC-45° (Nm)	157 ± 19	159 ± 30	159 ± 29	1.4%	1.4%	0.06%

BL: baseline, PLA: placebo, POMj: pomegranate juice, VJH: vertical jump height, HGS: handgrip strength, TMB: throwing medicine ball, BS: back-scratch, APT: absolute peak torque, RPT: relative peak torque, TPT: time to peak torque, AvRFD: average rate of force development, AvP: average power, MVIC: maximum voluntary isometric contraction, kg: kilogram, cm: centimeter, mm: millimeter s: second, Nm: Newton meter, ms: millisecond, and N/s: Newton per second.

**Table 4.** Comparison of functional tests, knee extensor and flexor isokinetic and isometric strength parameters, between the three conditions (*n *= 14).

Variables			PLA		POMj	
			BL	POMj	BL	PLA
Functional	VJH (cm)	MD	−1.0	−1.3	0.2	1.3
		Sig	0.772	0.632	1.000	0.632
		95% CI	−3.5 to 1.4	−4.1 to 1.4	−3.1 to 3.7	−1.4 to 4.1
	HGS (kg)	MD	3.2	−1.4	4.7	1.4
		Sig	1.000	1.000	1.000	1.000
		95% CI	−11.6 to 18.2	−10.0 to 7.1	−11.8 to 21.2	−7.1 to 10.0
	TMB (cm)	MD	−42.6	−34.0	−8.5	34.0
		Sig	0.463	0.728	1.000	0.728
		95% CI	−120.0 to 34.7	−110.5 to 42.3	−69.1 to 51.9	−42.3 to 110.5
	BS (mm)	MD	12.1	−1.4	13.5	1.4
		Sig	0.669	1.000	0.444	1.000
		95% CI	−1.3 to 3.8	−2.1 to 1.8	−1.0 to 3.7	−1.8 to 2.1
	V-seat (cm)	MD	1.2	−2.1	3.4	2.1
		Sig	0.896	0.470	0.024	0.470
		95% CI	−1.9 to 4.5	−6.0 to 1.7	0.4–6.4	−1.7 to 6.0
	Wall-squat (s)	MD	−7.5	−34.5	27.0	34.5
		Sig	1.000	0.374	1.000	0.374
		95% CI	−66.1 to 51.1	−92.3 to 23.2	−76.4 to 113.5	−23.2 to 92.3
Knee extensors	APT-30°/s (Nm)	MD	11.9	−2.1	14.1	2.1
		Sig	1.000	1.000	0.795	1.000
		95% CI	−31.0 to 54.9	−45.6 to 41.2	−19.1 to 47.3	−41.2 to 45.6
	APT-180°/s (Nm)	MD	17.3	−5.7	23.0	5.7
		Sig	0.323	1.000	0.002	1.000
		95% CI	−10.2 to 44.8	−25.4 to 13.9	9.1–37.0	−13.9 to 25.4
	RPT-30°/s (%)	MD	17.3	−7.1	24.5	7.1
		Sig	1.000	1.000	0.632	1.000
		95% CI	−45.8 to 80.5	−73.2 to 57.9	−26.5 to 75.5	−57.9 to 72.2
	RPT-180°/s (%)	MD	23.6	−11.1	34.7	11.1
		Sig	0.407	1.000	0.002	1.000
		95% CI	−17.1 to 64.5	−42.5 to 20.3	13.4–56.1	−20.3 to 42.5
	TPT-30°/s (ms)	MD	−103.5	45.0	−148.5	−45.0
		Sig	0.405	1.000	0.245	1.000
		95% CI	−282.0 to 74.8	−154.3 to 244.3	−364.6 to 67.5	−244.3 to 154.3
	TPT-180°/s (ms)	MD	−35.7	3.5	−39.2	−3.5
		Sig	0.581	1.000	0.232	1.000
		95% CI	−107.2 to 37.8	−53.3 to 60.4	−95.5 to 16.9	−60.4 to 53.3
	AvRFD-30°/s (N/s)	MD	0.07	−0.10	0.18	0.10
		Sig	0.269	0.851	0.099	0.851
		95% CI	−0.03 to 0.19	−0.36 to 0.15	−0.02 to 0.40	−0.15 to 0.36
	AvRFD-180°/s (N/s)	MD	0.12	−0.08	0.21	−0.08
		Sig	0.743	0.848	0.104	0.848
		95% CI	−0.16 to 0.42	−0.30 to 0.12	−0.03 to 0.47	−0.12 to 0.30
	AvP-30°/s (watts)	MD	15.2	−0.6	15.8	0.6
		Sig	0.006	1.000	0.001	1.000
		95% CI	4.4–25.9	−13.5 to 12.1	6.9–24.7	−12.1 to 13.5
	AvP-180°/s (watts)	MD	74.2	−0.8	75.1	0.8
		Sig	0.009	1.000	0.001	1.000
		95% CI	18.3–130.2	−52.5 to 50.8	43.9–106.3	−50.8 to 52.5
	MVIC-45° (Nm)	MD	10.2	−4.9	15.1	4.9
		Sig	0.698	1.000	0.083	1.000
		95% CI	−12.1 to 32.6	−25.6 to 15.8	−1.6 to 31.9	−15.8 to 25.6
Knee flexors	APT-30°/s (Nm)	MD	8.4	−0.3	8.8	0.3
		Sig	0.162	1.000	0.400	1.000
		95% CI	−2.5 to 19.4	−16.2 to 15.4	−6.3 to 24.0	−15.4 to 16.2
	APT-180°/s (Nm)	MD	16.1	−2.9	19.1	2.9
		Sig	0.045	1.000	0.015	1.000
		95% CI	0.2–32.5	−15.7 to 9.7	3.4–34.7	−9.7 to 15.7
	RPT-30°/s (%)	MD	13.3	−1.2	14.6	1.2
		Sig	0.452	1.000	0.163	1.000
		95% CI	−10.6 to 37.2	−26.3 to 23.8	−4.3 to 33.5	−23.8 to 26.3
	RPT-180°/s (%)	MD	23.2	−5.2	28.5	5.2
		Sig	0.072	1.000	0.015	1.000
		95% CI	−1.7 to 48.2	−23.6 to 13.1	5.2–51.8	−13.1 to 23.6
	TPT-30°/s (ms)	MD	−142.8	13.5	−156.4	−13.5
		Sig	0.349	1.000	0.174	1.000
		95% CI	−375.9 to 90.2	−318.7 to 345.8	−363.1 to 50.2	−345.8 to 318.7
	TPT-180°/s (ms)	MD	−45.0	16.4	−61.4	−16.4
		Sig	1.000	1.000	0.166	1.000
		95% CI	−179.7 to 89.7	−73.9 to 106.7	−141.5 to 18.6	−106.7 to 73.9
	AvRFD-30°/s (N/s)	MD	0.07	0.00	0.07	0.00
		Sig	0.033	1.000	0.013	1.000
		95% CI	0.01–0.13	−0.10 to 0.09	0.01–0.13	−0.09 to 0.10
	AvRFD-180°/s (N/s)	MD	0.26	−0.09	0.35	0.09
		Sig	0.047	1.000	0.011	1.000
		95% CI	0.01–0.53	−0.35 to 0.17	0.08–0.63	−0.17 to 0.35
	AvP-30°/s (watts)	MD	6.3	−0.2	6.5	0.2
		Sig	0.116	1.000	0.027	1.000
		95% CI	−0.6 to 11.9	−6.45 to 5.9	1.2–14.3	−5.9 to 6.4
	AvP-180°/s (watts)	MD	38.7	−9.3	48.1	9.3
		Sig	0.034	1.000	0.002	1.000
		95% CI	2.7–74.7	−35.0 to 16.4	19.0–77.1	−16.4 to 35.0
	MVIC-45° (Nm)	MD	2.2	−0.08	2.3	0.08
		Sig	1.000	1.000	1.000	1.000
		95% CI	−12.4 to 16.9	−11.4 to 11.2	−9.9 to 14.6	−11.2 to 11.4

BL: baseline, PLA: placebo, POMj: pomegranate juice, MD: mean difference, CI: confidence interval, VJH: vertical jump height, HGS: handgrip strength, TMB: throwing medicine ball, BS: back-scratch, APT: absolute peak torque, RPT: relative peak torque, TPT: time to peak torque, AvRFD: average rate of force development, AvP: average power, MVIC: maximum voluntary isometric contraction, kg: kilogram, cm: centimeter, mm: millimeter, s: second, Nm: Newton meter, ms: millisecond, and N/s: Newton per second.

**Functional tests:** A significant main effect was observed for the V-sit and reach (*F*₁,₈₁ = 3.88, *p *= 0.039, *ηp*² = 0.230). Post hoc analysis showed improvement from BL in the POMj condition (*p *= 0.024), with no between-condition differences (POMj vs. PLA) at the corresponding time points (BL: *p *= 0.896; post-EIMD: *p *= 0.470). For the remaining functional outcomes, the intervention effect was not significant: VJH (*F*₁,₇₂ = 0.88, *p *= 0.413, *ηp*² = 0.064), HGS (*F*₁,₄₃ = 0.46, *p *= 0.574, *ηp*² = 0.034), TMB (*F*₁,₈₄ = 1.48, *p *= 0.246, *ηp*² = 0.102), back-scratch (*F*₁,₈₃ = 1.53, *p *= 0.241, *ηp*² = 0.104), and wall-squat (*F*₁,₁₇ = 0.85, *p *= 0.388, *ηp*² = 0.062) (Figure 3, Table 4).

**Knee extensor isokinetic and isometric parameters**: For knee extensor outcomes, the repeated-measures analysis showed a significant main effect of the intervention for APT-180° s^−1^ (*F*₁,₂₉ = 4.87, *p* = 0.034, *ηp*² = 0.273), with post-hoc tests indicating a POMj > BL improvement (*p *= 0.002) but no differences vs. PLA at BL (*p *= 0.323) or post-EIMD (*p *= 1.000) (Figure 4, Table 4). For RPT-180° s^−1^, the main effect was also significant (*F*₁,₃₇ = 4.59, *p *= 0.036, *ηp*² = 0.261); Bonferroni tests showed POMj > BL (*p *= 0.002), with no differences between POMj and PLA (*p *= 1.000) or between PLA and BL (*p *= 0.407) (Figure 4, Table 4). AvP-30° s^−1^ displayed a significant main effect (*F*₁,₇₀ = 10.14, *p *= 0.001, *ηp*² = 0.438), where both PLA (*p *= 0.006) and POMj (*p *= 0.001) exceeded BL, yet POMj vs. PLA remained nonsignificant (*p*=1.000) (Figure 4, Table 4). Likewise, AvP-180° s^−1^ showed a significant main effect (*F*₁,₄₉ = 12.41, *p *= 0.001, *ηp*² = 0.489), with increases vs. BL in PLA (*p *= 0.009) and POMj (*p *= 0.001) but no difference in the POMj–PLA (*p *= 1.000) (Figure 4, Table 4). No significant intervention effects emerged for APT-30° s^−1^ (*F*₁,₈₁ = 0.53, *p *= 0.574, *ηp*² = 0.040), RPT-30° s^−1^ (*F*₁,₈₄ = 0.65, *p *= 0.517, *ηp*² = 0.048), TPT-30° s^−1^ (*F*₁,₉₁ = 2.22, *p *= 0.132, *ηp*² = 0.146), TPT-180° s^−1^ (*F*₁,₈₀ = 1.85, *p *= 0.181, *ηp*² = 0.125), AvRFD-30° s^−1^ (*F*₁,₂₉ = 3.06, *p *= 0.090, *ηp*² = 0.191), AvRFD-180° s^−1^ (*F*₁,₇₉ = 2.75, *p *= 0.090, *ηp*² = 0.175), and MVIC-45° (*F*₁,₈₁ = 2.23, *p *= 0.133, *ηp*² = 0.147) (Figure 4, Table 4).

**Knee flexor isokinetic and isometric parameters**: For knee flexor outcomes, the intervention showed a significant main effect for APT-180° s^−1^ (*F*₁,₈₅ = 7.06, *p *= 0.005, *ηp*² = 0.352); post-hoc tests indicated increases vs. BL in both PLA (*p *= 0.045) and POMj (*p *= 0.015), with no POMj–PLA difference (*p *= 1.000) (Figure 5, Table 4). RPT-180° s^−1^ also showed a significant main effect (*F*₁,₇₉ = 6.92, *p *= 0.005, *ηp*² = 0.348); Bonferroni tests revealed POMj > BL (*p *= 0.015) with no differences between POMj and PLA (*p *= 1.000) or between PLA and BL (*p *= 0.072) (Figure 5, Table 4). For AvRFD-30° s^−1^, the main effect was significant (*F*₁,₃₇ = 4.53, *p *= 0.037, *ηp*² = 0.259); both PLA (*p *= 0.033) and POMj (*p *= 0.013) exceeded BL, with no POMj–PLA difference (*p *= 1.000) (Figure 5, Table 4). AvRFD-180° s^−1^ likewise demonstrated a significant main effect (*F*₁,₉₈ = 7.24, *p *= 0.003, *ηp*² = 0.358); post hoc tests showed increases vs. BL in PLA (*p *= 0.047) and POMj (*p *= 0.011), again  with no POMj–PLA difference (*p *= 1.000) (Figure 5, Table 4). AvP-30° s^−1^ displayed a significant main effect (*F*₁,₇₂ = 4.72, *p *= 0.023, *ηp*² = 0.267); POMj > BL (*p *= 0.027), while POMj vs. PLA (*p *= 1.000) and PLA vs. BL (*p *= 0.116) were not different (Figure 5, Table 4). AvP-180° s^−1^ showed a significant main effect (*F*₁,₇₂ = 10.48, *p *= 0.001, *ηp*² = 0.446); both PLA (*p *= 0.034) and POMj (*p*=0.002) improved vs. BL, with no difference in POMj–PLA (*p *= 1.000) (Figure 5, Table 4). No significant intervention effects were observed for APT-30° s^−1^ (*F*₁,₇₂ = 1.88, *p *= 0.179, *ηp*² = 0.127), RPT-30° s^−1^ (*F*₁,₈₁ = 1.89, *p *= 0.176, *ηp*² = 0.127), TPT-30° s^−1^ (*F*₁,₄₆ = 1.63, *p *= 0.221, *ηp*² = 0.112), TPT-180° s^−1^ (*F*₁,₃₈ = 1.39, *p *= 0.265, *ηp*² = 0.097), or MVIC-45° (*F*₁,₈₂ = 0.16, *p *= 0.833, *ηp*² = 0.012) (Figure 5, Table 4).

**Delayed-onset muscle soreness**: There was a significant main effect of time (*F*₂,₈₇ = 27.15, *p *= 0.001, *ηp*² = 0.676), with no time × condition interaction (*F*₂,₃₂ = 0.28, *p *= 0.787, *ηp*² = 0.021). Bonferroni-adjusted comparisons showed that, in PLA, DOMS at 0h, 12, and 24 h were higher than BL (*p *= 0.001, 0.001, and 0.003). In POMj, DOMS at 0, 12, 24, and 48 h exceeded BL (*p *= 0.001, 0.001, 0.001, 0.049, respectively). Between-condition differences were not significant at any time point—BL, 0, 12, 24, and 48 h (all *p *> 0.05). See Figure 6 and Table 5 for complete statistics.

**Table 5.** Means, standard deviations (SD), and relative changes (%RC) of DOMS for PLA and POMj (*n *= 14).

Time	PLA (mm)	POMj (mm)	%RC_POMj/PLA_
	Mean ± SD	Mean ± SD	
Baseline	17.8 ± 18.0	12.1 ± 19.2	−32.0%
0 h	63.2 ± 25.5	60.7 ± 22.6	−3.9%
12 h	48.5 ± 17.9	46.4 ± 24.6	−4.3%
24 h	46.4 ± 25.9	45.7 ± 20.3	−1.5%
48 h	40.3 ± 24.9	37.1 ± 28.6	−7.9%

PLA: placebo, POMj: pomegranate juice, Baseline: before the EIMD; 0 h: immediately after the EIMD; 12 h: 12 hours after the EIMD; 24 h: 24 hours after the EIMD; 48 h: 48 hours after the EIMD; and mm: millimeter.

**SWC Analysis:** In the SWC analysis (SWC = 0.2 × SD at BL), the functional tests showed heterogeneous but practically meaningful individual improvements (Table 6). VJH responders were 35.7% in the POMj condition vs. 28.6% in PLA, with 28.6% classified as co-responders (responding to both conditions). HGS showed identical responder proportions for POMj and PLA (50.0% in each), with 35.7% of the patients being co-responders. For TMB, 35.7% of the athletes were responders under POMj compared with 21.4% under PLA, and 7.1% were co-responders. BS yielded 42.9% of responders with POMj and 50.0% of responders with PLA (co-responders 35.7%), whereas V-SIT demonstrated 57.1% of responders in both conditions, with 42.9% of co-responders. Wall-squat exhibited 28.6% of the responders with POMj vs. 21.4% with PLA, and 21.4% were co-responders. For knee extensors, responder proportions favored POMj on key high-velocity indices: for RPT-180° s^−1^, 85.7% of athletes were responders with POMj vs. 64.3% with PLA (co-responders 57.1%), and for APT-180° s^−1^, 78.6% vs. 71.4% (co-responders 64.3%), respectively. Other knee extensor measures were broadly comparable between conditions: APT-30° s^−1^ and RPT-30° s^−1^ each showed 64.3% of responders in both POMj and PLA (co-responders 64.3%); TPT-30° s^−1^ 78.6% (POMj) vs. 57.1% (PLA) (co-responders 42.9%); TPT-180° s^−1^ 57.1% vs. 57.1% (co-responders 50.0%); AvRFD-30° s^−1^ 64.3% vs. 64.3% (co-responders 42.9%); AvRFD-180° s^−1^ 78.6% vs. 78.6% (co-responders 71.4%); and AvP-30° s^−1^ 78.6% vs. 71.4% (co-responders 64.3%). AvP-180° s^−1^ similarly showed high responder rates under both conditions, with no clear advantage for either trial. For the knee flexors, the proportion of responders also favored the POMj condition in high-velocity measures. For RPT-180° s^−1^, 85.7% of the athletes were responders to POMj, compared with 57.1% with PLA. Similarly, for APT-180° s^−1^, the responder rate was 78.5% for POMj versus 64.2% for PLA. For low-velocity measures (30° s^−1^), responder rates were more balanced, with 64.2% responding for both APT and RPT under both conditions. Taken together, these athlete-level data indicate that although group-mean differences between POMj and PLA were limited, POMj elicited a greater proportion of responders for selected high-velocity outcomes in both the knee extensors and flexors. At the same time, response patterns across most low-velocity and isometric variables remained broadly similar between conditions (Table 6).

**Table 6.** Individual-level performance changes based on SWC analysis across BL, PLA, and POMj conditions (*n *= 14).

	Variables	SWC (0.2 × SD_BL_)	Responders PLA (*n*)	Responders POMj (*n*)	Co-responders PLA and POMj (*n*)	Responders PLA (%)	Responders POMj (%)	Co-responders PLA and POMj (%)
Functional	VJH (cm)	1.62	4	5	4	28.5%	35.7%	28.5%
	HGS (kg)	4.66	7	7	5	50.0%	50.0%	35.7%
	TMB (cm)	22.64	3	5	1	21.4%	35.7%	7.1%
	BS (mm)	12.5	7	6	5	50.0%	42.8%	35.7%
	V-seat (cm)	1.94	8	8	6	57.1%	57.1%	42.8%
	Wall-sit (s)	19.71	3	4	3	21.4%	28.5%	21.4%
Knee extensors	APT-30°/s (Nm)	7.68	9	9	9	64.2%	64.2%	64.2%
	APT-180°/s (Nm)	5.84	10	11	9	71.4%	78.5%	64.2%
	RPT-30°/s (%)	12.83	9	9	9	64.2%	64.2%	64.2%
	RPT-180°/s (%)	8.02	9	12	8	64.2%	85.7%	57.1%
	TPT-30°/s (ms)	61.43	8	11	6	57.1%	78.5%	42.8%
	TPT-180°/s (ms)	20.15	8	8	7	57.1%	57.1%	50.0%
	AvRFD-30°/s (N/s)	0.04	9	9	6	64.2%	64.2%	42.8%
	AvRFD-180°/s (N/s)	0.05	11	11	10	78.5%	78.5%	71.4%
	AvP-30°/s (watts)	3.79	10	11	9	71.4%	78.5%	64.2%
	AvP-180°/s (watts)	11.84	12	13	12	85.7%	92.8%	85.7%
	MVIC-45° (Nm)	6.91	7	9	5	50.0%	64.2%	35.7%
Knee flexors	APT-30°/s (Nm)	4.11	8	10	7	57.1%	71.4%	50.0%
	APT-180°/s (Nm)	4.02	10	10	8	71.4%	71.4%	57.1%
	RPT-30°/s (%)	6.81	9	6	4	64.2%	42.8%	28.5%
	RPT-180°/s (%)	6.49	10	10	8	71.4%	71.4%	57.1%
	TPT-30°/s (ms)	60.47	8	10	6	57.1%	71.4%	42.8%
	TPT-180°/s (ms)	23.63	8	8	6	57.1%	57.1%	42.8%
	AvRFD-30°/s (N/s)	0.01	8	12	6	57.1%	85.7%	42.8%
	AvRFD-180°/s (N/s)	0.05	10	9	8	71.4%	64.2%	57.1%
	AvP-30°/s (watts)	1.84	10	10	7	71.4%	71.4%	50.0%
	AvP-180°/s (watts)	8.98	10	12	9	71.4%	85.7%	64.2%
	MVIC-45° (Nm)	3.80	6	7	4	42.8%	50.0%	28.5%

SWC: smallest worthwhile change, BL: baseline, PLA: placebo, POMj: pomegranate juice, VJH: vertical jump height, HGS: handgrip strength, TMB: throwing medicine ball, BS: back-scratch, APT: absolute peak torque, RPT: relative peak torque, TPT: time to peak torque, AvRFD: average rate of force development, AvP: average power, MVIC: maximum voluntary isometric contraction, kg: kilogram, cm: centimeter, mm: millimeter, s: second, Nm: Newton meter, ms: millisecond, and N/s: Newton per second.

## 4. Discussion

This double-blind crossover study examined whether short-term POMj supplementation enhances functional recovery in collegiate male volleyball players. Overall, our results indicate that POMj did not significantly outperform placebo on group-average recovery outcomes at 48 h post-EIMD. Both the POMj and placebo conditions showed comparable improvements from baseline in strength (isokinetic and isometric knee measures) and functional performance. Additionally, DOMS increased and subsequently declined with a similar time course in both trials. These findings suggest that, in this well-trained population, the mean effect of POMj on conventional functional recovery metrics was modest. However, an important nuance emerged when responses were examined at the individual level: SWC analysis demonstrated that a higher proportion of athletes achieved practically meaningful improvements with POMj for specific high-velocity knee extensor indices. For example, for RPT-180° s^−1^, 85.7% of players exceeded the SWC threshold in the POMj condition compared with 64.3% in the placebo condition, and a similar tendency was evident for APT-180° s^−1^ (78.6% vs. 71.4%). These data indicate that although average changes were small, a subset of athletes derived substantial and worthwhile performance benefits from POMj. In practical terms, this pattern implies that POMj did not uniformly accelerate recovery across all players. Still, individual response heterogeneity suggests that certain athletes may derive meaningful benefits, likely influenced by factors such as training status, baseline recovery capacity, and the degree of muscle damage incurred.

Compared with previous research, our findings contribute to the growing yet mixed body of research on pomegranate supplementation for exercise recovery. Initial studies on POMj in untrained or recreational individuals often reported clear benefits, such as faster strength recovery and reduced soreness after intense eccentric exercise [16,20]. For example, consuming natural POMj for several days around heavy resistance exercise has been shown to increase antioxidant capacity and attenuate oxidative damage, resulting in quicker restoration of muscle strength compared to a PLA [55]. In addition, Trombold et al. found that POMj supplementation improved strength recovery 2–3 days postexercise and reduced muscle soreness in strength-trained men [16]. Similarly, Ammar et al. reported accelerated recovery of strength and soreness markers following a weightlifting training session with POMj [8]. Such outcomes support the notion that the rich polyphenol content of pomegranate can mitigate EIMD by blunting excessive inflammation and oxidative stress [15,17]. Mechanistically, the antioxidant and anti-inflammatory effects of POMj are thought to protect muscle fibers from secondary damage, which helps preserve force-generating capacity during recovery [7,17]. Another proposed mechanism is enhanced muscle perfusion: acute pomegranate intake can increase blood vessel diameter and blood flow postexercise, likely via improved nitric oxide bioavailability [18]. This vasodilatory response could facilitate nutrient delivery and waste removal in recovering muscle, potentially accelerating the repair process [18]. These collective findings in the literature portray POMj as a promising natural intervention to accelerate recovery through both antioxidant and circulatory pathways [7].

Despite this encouraging background, not all studies have observed uniform benefits with POMj, and our results align with those reporting more nuanced or limited effects. Trombold et al., for instance, found that POMj improved recovery in elbow flexors but not in knee extensors in resistance-trained men [17]. In that study, compared with placebo, POMj significantly accelerated the recovery of elbow flexor strength and reduced arm soreness, whereas knee extensor strength and soreness remained unchanged [17]. This muscle-group discrepancy highlights that the efficacy of POMj may depend on the context, including the muscles involved and the nature of the exercise. Our trial similarly found no difference in soreness between POMj and PLA, which aligns with specific reports that show strength improved with POMj, but subjective DOMS did not [21]. Such inconsistencies are noted in the literature: a recent systematic review and meta-analysis concluded that pomegranate supplementation does not markedly enhance overall markers of muscle damage. However, there is evidence of small, short-term benefits in muscle function recovery [21]. Indeed, the meta-analysis emphasized that the most apparent benefits of POMj have been observed so far in resistance-based EIMD models, while evidence in endurance or sport-specific protocols is still emerging and less conclusive. Our findings—showing no clear group-level advantage of POMj in this volleyball-specific recovery context—therefore contribute valuable nuance to this developing area, suggesting that POMj's effects in high-level sports may be more subtle and context-dependent and may be best captured through individualized rather than purely group-based analyses.

Several factors likely explain why POMj did not significantly outperform placebo at the group level in our study. First, our participants were highly trained athletes, and prior research suggests that well-trained individuals experience a blunted damage response to exercise, making it harder to detect supplementation effects unless the stress is sufficiently severe [22]. In our protocol, some recovery occurred naturally by 48 h in the PLA condition, implying that the EIMD stimulus—while functionally meaningful—may not have been detrimental enough for the athletes' innate recovery capacity. In contrast, many positive POMj studies have used untrained subjects or isolated eccentric exercises that induce greater muscle damage. Most POMj research to date has been in laboratory or single-joint exercise models with untrained or recreational participants. By studying collegiate volleyball players performing sport-specific, multijoint actions, we addressed a critical gap; however, this real-world context involves a higher baseline adaptability. It is notable that our athletes still recovered strength within 48 h, even on a PLA, reflecting the robust recovery ability of trained muscles. Second, the dosing regimen and timing of POMj supplementation might influence outcomes. We provided ~1000 mL of POMj split between the evening before and 2 h pre-EIMD, which is a relatively acute protocol. In contrast, some studies showing substantial benefits employed multiday loading of POMj before and after the damaging exercise [8,15]. Effective protocols in the literature often span ~5 days of twice-daily POMj around the exercise. It is possible that extending POMj intake for several days postexercise (to continue supplying polyphenols during the recovery window) would amplify its effects. Our trial prioritized a practical preloading strategy, which aligns with many real-world applications. However, future work may explore whether longer or postexercise POMj supplementation yields greater improvements. Finally, interindividual variability must be considered, as evidenced by our SWC findings; some athletes responded better than others. This variability can dilute group mean differences and has been observed with vitamin C supplementation as well [25].

Beyond pomegranate, our results resonate with findings on other polyphenol-rich interventions for recovery. Polyphenols in general have attracted attention for mitigating EIMD because of their antioxidant and anti-inflammatory properties [7]. For example, tart cherry juice—rich in anthocyanins—is well documented to reduce muscle soreness and improve strength recovery in athletes, much like POMj. A recent systematic review by Vitošević et al. compared several fruit juices and found that pomegranate and tart cherry juices were consistently effective in decreasing postexercise soreness and inflammatory marker levels, particularly when consumed over multiple days [10]. These natural juices appear to support recovery without the side effects associated with high-dose pharmacological antioxidants. However, their performance benefits are usually modest and context-dependent [10]. Our observation that POMj conferred subtle improvements in explosive strength for some athletes aligns with broader evidence that polyphenol supplementation can aid recovery but may not substantially enhance performance unless a specific deficit is present. In contrast to our study on pomegranate, purple grape juice—another polyphenol-rich beverage—has shown promise in other athletic populations. For example, in young elite soccer players, supplementation with grape juice improved specific endurance and power metrics following intense exercise [27]. Notably, in that study, the group-average effects were minor, yet many individuals experienced meaningful improvements—a scenario very similar to our POMj findings [27]. Taken together, the literature suggests that polyphenol nutraceuticals (such as pomegranate, cherry, and grape) offer a useful, albeit modest, recovery boost, particularly in reducing soreness and helping muscles regain strength more quickly. Our results temper this enthusiasm by highlighting that in high-performance athletes, these benefits may be challenging to detect at the group level, reinforcing the importance of personalized evaluation.

### 4.1. Individual response variability and SWC analysis

The SWC analysis provides an essential complementary perspective to the null findings at the group level, showing that many athletes experienced practically meaningful performance improvements, even when the mean differences between POMj and PLA were minor. By defining responders as those whose change exceeded 0.2 × SD at baseline, we focused on improvements that are likely to be “felt” by athletes and coaches rather than representing trivial day-to-day noise [24]. In the functional domain, between ~21% and 57% of players exceeded the SWC threshold in each test under both conditions, with slightly higher responder proportions for POMj in several outcomes. For example, 35.7% of athletes were classified as responders in VJH and 35.7% in the medicine ball throw with POMj, compared with 28.6% and 21.4% in the PLA condition, respectively; wall squats likewise showed a numerically higher proportion of responders with POMj (28.6% vs. 21.4%). In contrast, handgrip strength, back-scratch, and V-sit flexibility displayed broadly similar responder rates across conditions, with a substantial proportion of athletes acting as co-responders. This suggests that part of the improvement likely reflects training adaptation, familiarization, or natural recovery from the initial damage stimulus rather than a pure supplement effect. The clearest SWC signal in favor of POMj emerged for high-velocity knee extensor indices: for APT-180° s^−1^ and RPT-180° s^−1^, 78.6% and 85.7% of players, respectively, exceeded the SWC threshold with POMj, compared with 71.4% and 64.3% under PLA, with more than half of the cohort classified as co-responders in these variables. These patterns are consistent with the ANOVA results, which show significant within-condition improvements at 180° s^−1^ in the POMj arm. However, they add nuance by indicating that, from an athlete-centered perspective, the probability of achieving a worthwhile gain in high-velocity force production was modestly higher with POMj than with the placebo. Importantly, responder rates were high for many extensor and flexor parameters in both conditions (often ≥50%), underscoring that the EIMD protocol, combined with ongoing training, created a strong recovery and adaptation stimulus; within this generally favorable context, POMj appears to shift the distribution slightly toward more athletes achieving changes beyond the SWC for the fastest, most sport-relevant contractions rather than producing a large, uniform shift in group means.

These findings align closely with previous SWC-based work from our group, in which substantial proportions of “responders” were identified despite minor or nonsignificant mean effects. In strength-trained men, caffeine supplementation yielded sizeable responder rates on key performance variables, even when traditional statistics suggested modest average benefits [26]. Similarly, in elite soccer players, a polyphenol-rich juice led many individuals to exceed SWC thresholds in endurance and explosive-power outcomes, while group-level differences remained limited [27]. Together with the present POMj data, these studies support the idea that recovery-oriented nutraceuticals may primarily increase the likelihood that a given athlete experiences a worthwhile improvement rather than guaranteeing a large mean effect across an already well-trained group. From a practical perspective, this challenges the “average athlete” view and reinforces that decisions about POMj use should consider individual response patterns, possibly through structured N-of-1 trials or short monitoring blocks integrated into the training process.

**Limitations:** This study has several limitations that should be considered when interpreting the findings. First, the sample size was modest (*n *= 14), although the randomized, double-blind crossover design partly mitigates this by allowing each athlete to serve as his own control. Second, recovery was assessed only at 48 h post-EIMD; any earlier or later effects of POMj (e.g. at 24 or 72 h) may have been missed. Third, although the EIMD protocol was sport-specific and ecologically valid, it may not have produced severe muscle damage in these highly trained players, reducing the scope for a detectable supplementation effect. Fourth, a repeated-bout effect cannot be entirely excluded: despite the 7-day washout and counterbalanced order, performing the damaging protocol twice may have attenuated muscle damage during the second period in both conditions. Fifth, we did not assess biochemical markers of muscle damage or inflammation, which limits mechanistic insight into how POMj may influence recovery. Additionally, the sample consisted only of young male collegiate volleyball players, which limits the generalizability of the results to female athletes, athletes in other age groups, and participants in different sports. Finally, the present trial used a relatively short pre-exercise loading strategy (1000 mL POMj consumed only on the evening before and two h before EIMD); several studies reporting more apparent benefits of polyphenol-rich juices have employed multi-day pre- and/or postexercise loading protocols, so future research should examine whether longer-term POMj supplementation around training yields greater or more consistent recovery benefits.

### 4.2. Conclusion and practical guidelines

In this double-blind crossover study on collegiate male volleyball players, short-term POMj supplementation did not produce clear group-level benefits over placebo for recovery 48 h after EIMD. The functional tests, isokinetic/isometric knee strength tests, and DOMS tests followed similar patterns under both conditions, indicating that the average recovery effect of POMj was modest in this well-trained population. However, SWC analysis showed that a higher proportion of athletes achieved practically meaningful improvements in selected high-velocity knee extensor outcomes (e.g. peak torque at 180°/s) with POMj than with placebo, suggesting that some individuals can derive worthwhile performance gains.

Practically, POMj should be seen as a supportive recovery aid rather than a transformative one, benefiting some athletes more than others. Coaches can trial ~1000 mL of natural POMj (500 mL the evening before and 500 mL ~2 h before intense sessions) alongside core recovery methods. Monitoring jump height, isokinetic performance, and soreness over a few weeks can help identify effective responses. For athletes who show no consistent improvement, the focus should shift to other individualized interventions.

## Data Availability

The datasets used and/or analyzed during the current study are available from the corresponding author upon reasonable request.
